# Tibetans living at sea level have a hyporesponsive hypoxia-inducible factor system and blunted physiological responses to hypoxia

**DOI:** 10.1152/japplphysiol.00535.2013

**Published:** 2013-09-12

**Authors:** Nayia Petousi, Quentin P. P. Croft, Gianpiero L. Cavalleri, Hung-Yuan Cheng, Federico Formenti, Koji Ishida, Daniel Lunn, Mark McCormack, Kevin V. Shianna, Nick P. Talbot, Peter J. Ratcliffe, Peter A. Robbins

**Affiliations:** ^1^Department of Physiology, Anatomy and Genetics, University of Oxford, Oxford, United Kingdom;; ^2^Molecular and Cellular Therapeutics, The Royal College of Surgeons in Ireland, Dublin, Ireland;; ^3^Nuffield Department of Clinical Medicine, University of Oxford, Oxford, United Kingdom;; ^4^Research Center of Health, Physical Fitness and Sports, Nagoya University, Nagoya, Japan;; ^5^Department of Statistics, University of Oxford, Oxford, United Kingdom; and; ^6^Duke University, Durham, North Carolina

**Keywords:** hypoxic pulmonary vasoconstriction, *EPAS1*, *EGLN1*, erythropoietin, high altitude

## Abstract

Tibetan natives have lived on the Tibetan plateau (altitude ∼4,000 m) for at least 25,000 years, and as such they are adapted to life and reproduction in a hypoxic environment. Recent studies have identified two genetic loci, *EGLN1* and *EPAS1*, that have undergone natural selection in Tibetans, and further demonstrated an association of *EGLN1*/*EPAS1* genotype with hemoglobin concentration. Both genes encode major components of the hypoxia-inducible factor (HIF) transcriptional pathway, which coordinates an organism's response to hypoxia. Patients living at sea level with genetic disease of the HIF pathway have characteristic phenotypes at both the integrative-physiology and cellular level. We sought to test the hypothesis that natural selection to hypoxia within Tibetans results in related phenotypic differences. We compared Tibetans living at sea level with Han Chinese, who are Tibetans' most closely related major ethnic group. We found that Tibetans had a lower hemoglobin concentration, a higher pulmonary ventilation relative to metabolism, and blunted pulmonary vascular responses to both acute (minutes) and sustained (8 h) hypoxia. At the cellular level, the relative expression and hypoxic induction of HIF-regulated genes were significantly lower in peripheral blood lymphocytes from Tibetans compared with Han Chinese. Within the Tibetans, we found a significant correlation between both *EPAS1* and *EGLN1* genotype and the induction of erythropoietin by hypoxia. In conclusion, this study provides further evidence that Tibetans respond less vigorously to hypoxic challenge. This is evident at sea level and, at least in part, appears to arise from a hyporesponsive HIF transcriptional system.

the tibetan plateau is one of the highest regions on Earth. It has an average elevation of ∼4,000 m, a barometric pressure of <500 mmHg, and an ambient partial pressure of oxygen (Po_2_) of 80 mmHg. This low environmental Po_2_, termed hypobaric hypoxia, presents a significant physiological challenge to resident humans and is likely to have acted as an agent of natural selection. Humans have lived on this plateau for at least 25,000 years (25), which suggests that Tibetan natives will have had more time and opportunity for natural selection to occur in response to a hypoxic environment than any other high-altitude human population.^1^

Tibetan highlanders have distinct physiological characteristics and are resistant to diseases of high altitude. Compared with other high-altitude residents such as Andeans or Han Chinese migrants to high altitude, Tibetan highlanders ventilate more (6) and have lower hemoglobin concentrations (4, 17, 27, 42); for example, at 4,000 m Tibetans were found to have hemoglobin concentrations comparable with those of US sea-level residents (4). Tibetans also have a very low prevalence of chronic mountain sickness, a disease characterized by hypoventilation, pulmonary hypertension and excessive erythrocytosis (26). Despite the well-characterized physiological traits of Tibetans at high altitude, relatively little is known about the molecular or genetic mechanisms that may underlie these traits and whether they are preserved in Tibetans living at sea level.

Recent genome-wide studies comparing Tibetan with predominantly Han Chinese natives have found evidence of positive natural selection across a number of genomic regions (5, 8, 29, 36, 43, 44). These regions include the *EPAS1* locus, which encodes HIF2α, a transcription factor of the hypoxia-inducible factor (HIF) family that regulates gene expression in response to hypoxia, and the *EGLN1* locus, which encodes PHD2, an oxygen-dependent hydroxylase that contributes to the degradation of HIFs. These findings implicate the HIF pathway in the genetic adaptation of Tibetans to high altitude. Some of these reports demonstrated a correlation of genetic variation in these genes with hemoglobin concentration, such that among Tibetan natives, the putative selected haplotype in each gene is associated with lower hemoglobin concentration at high altitude (5, 36, 44).

The HIF pathway regulates not only hemoglobin concentration (34) but also many other aspects of physiology. This is evident from studies of patients with genetic diseases of the HIF pathway, including Chuvash polycythemia, in which individuals are homozygous for a hypomorphic allele of the von Hippel-Lindau (VHL) tumor suppressor, which impairs HIF degradation (2), and HIF2α gain-of-function mutations (30, 31). Apart from erythrocytosis, these patients were shown to have pulmonary hypertension and increased ventilatory and pulmonary vascular sensitivities to acute hypoxia when compared with healthy controls (15, 38).

In this study, we set out to examine, at sea level, the integrative physiology of Tibetan natives and to compare it with that of their most closely related major ethnic group, the Han Chinese. In particular we sought to determine whether Tibetans possess a distinct cardiopulmonary phenotype, as has been observed for patients with disease of the HIF system. We recruited 14 Tibetan and 13 Han Chinese volunteers living in the UK. After a set of baseline measurements, we determined their cardiopulmonary responses to acute (minutes) and sustained (8 h) hypoxia. To explore the existence of an intermediate phenotype affecting the HIF system, we also compared Tibetan and Han Chinese responses to hypoxia at the cellular level by examining the expression of HIF-regulated genes in peripheral blood lymphocytes (PBLs) cultured at graded oxygen tensions. In view of the reports of an association of putative *EGLN1* and *EPAS1* haplotypes with hemoglobin in Tibetan highlanders (5, 36), we also sought to define genotype-phenotype relationships for these haplotypes in the Tibetans that participated in our study.

## MATERIALS AND METHODS

### 

#### Participants.

Fourteen healthy male Tibetan volunteers and 13 healthy male Han Chinese volunteers (control group) participated in the study. Not all volunteers participated in all protocols of the study, as explained in the caption of Table 2. All Tibetan volunteers were born and had lived for a variable number of years (at least 6) on the Tibetan plateau before emigrating to sea level, where they all had lived for at least 4 years. Four volunteers had lived on the Tibetan plateau for less than 10 years, seven volunteers lived on the plateau between 10 and 20 years, and three volunteers had lived there for more than 20 years. After moving to sea level, two volunteers had lived at sea level for less than 10 years, ten volunteers between 10 and 20 years, and two volunteers for more than 20 years prior to being studied. All Han Chinese volunteers were sea-level residents. The study was explained to the volunteers both verbally and in writing, and each volunteer gave informed written consent before participating. The study was approved by the Berkshire Clinical Research Ethics Committee and performed in accordance with the Declaration of Helsinki.

#### Physiology protocols.

The physiological experiments took place in our laboratory in the following order. Each volunteer reported at 7:30 A.M. After a period of rest (∼20 min), his baseline end-tidal partial pressure of CO_2_ (Pet_CO_2__) was recorded while sitting quietly for 10 min. Thereafter, the volunteer undertook three acute hypoxic protocols, with a period of 10–15 min rest between protocols.

*Protocol 1* measured the acute hypoxic ventilatory response. During this protocol, the volunteer was seated upright and breathed through a mouthpiece with the nose occluded. The end-tidal partial pressure of oxygen (Pet_O_2__) was held at 100 mmHg (euoxia) for the first 5 min and this was followed by six square waves in Pet_O_2__ varying between 1 min at 50 mmHg (hypoxia) and 1 min at 100 mmHg. Pet_CO_2__ was held constant at 2 mmHg above the volunteer's resting value (isocapnia). *Protocol 2* measured the pulmonary vascular response to acute isocapnic hypoxia. The volunteer laid on a couch in the left lateral position and breathed through a mouthpiece. The gas mixture breathed varied so that Pet_O_2__ was held at 100 mmHg for the first 5 min, 50 mmHg for the next 10 min, and 100 mmHg for the final 10 min. Pet_CO_2__ was held constant at 2 mmHg above the volunteer's resting value. The pulmonary vascular response was assessed by noninvasive echocardiography throughout the protocol. *Protocol 3* measured the response of cardiac output to acute isocapnic hypoxia and was identical to *Protocol 2* except that echocardiography was used to determine cardiac output.

Following *Protocols 1–3* and after a 20-min rest period, the volunteer was exposed to 8 h of sustained isocapnic hypoxia in a purpose-built chamber. Pet_O_2__ was held at 50 mmHg and Pet_CO_2__ at the volunteer's normal air-breathing value using the technique of end-tidal forcing, as described below. Blood samples were taken to measure plasma erythropoietin at 0, 4, and 8 h. Following the chamber exposure and after a 30-min rest period, the volunteer repeated *Protocols 1*, *2*, and *3*. This marked the end of the experimental day. Throughout all protocols, the volunteer were continuously monitored by pulse oximetry and a three-lead ECG.

#### Control of end-tidal gases.

During the acute hypoxic exposures (*Protocols 1*, *2*, and *3*), respired gases were sampled via a fine catheter close to the mouth and analyzed continuously for partial pressures of carbon dioxide and oxygen (Pco_2_ and Po_2_, respectively) by mass spectrometry; end-tidal partial pressures of oxygen and carbon dioxide and oxygen (Pet_CO_2__ and Pet_O_2__, respectively) were detected in real time by a computer and logged breath by breath. A dynamic end-tidal forcing system (33) was used to control the end-tidal gases as specified in each protocol. Before the start of each experiment, a cardiorespiratory model was used to construct a forcing function that contained the breath-by-breath predicted values for inspiratory Pco_2_ and Po_2_ to produce the desired end-tidal gas sequences (33). During the experiment, the measured end-tidal partial pressures were compared with the desired values and a computer-controlled gas-mixing system delivered the appropriate concentrations of inspired O_2_, CO_2_, and N_2_ to achieve the desired values, using feedback control.

During the chamber hypoxic exposure, respired gas was sampled via nasal cannulae and analyzed using a commercial CO_2_/O_2_ gas analyzer (Datex Normocap 200 Oxy; GE Healthcare, UK) and the values were input to a computer. Pet_CO_2__ and Pet_O_2__ were detected and recorded breath-by-breath. The computer automatically adjusted the composition of the gas in the chamber every 5 min, or at manually overridden intervals, to minimize the error between desired and actual values for the end-tidal gases. This system has been described in detail elsewhere (20).

#### Echocardiography.

Pulmonary arterial systolic pressure (PASP) and cardiac output (Q̇) were estimated through Doppler echocardiography (Vivid-i; GE Healthcare, Little Chalfont, UK).

Most people have detectable regurgitation through their tricuspid valve during systole. The peak pressure difference (ΔPmax) across the tricuspid valve was calculated using the modified Bernoulli's equation where ΔPmax = ρυ^2^/2, where ρ is the density of blood and υ is the measured peak velocity of the tricuspid regurgitation jet. PASP was then calculated as PASP = RAP + ΔPmax, where RAP is the right atrial pressure. In this study, RAP was assumed constant at 5 mmHg and unaffected by hypoxia (19), and thus changes in ΔPmax mirror changes in PASP, and are a measure of pulmonary vascular response to hypoxia. This technique has been extensively validated (1, 12, 28, 37, 45). Up to five PASP measurements per minute of *Protocol 2* were recorded. PASP under conditions of euoxia (PASP_euoxia_) and PASP under conditions of hypoxia (PASP_hypoxia_) were calculated for each volunteer by averaging all PASP measurements noted in the first 5 min of isocapnic euoxia and in the last 6 min of isocapnic hypoxia of *Protocol 2*, respectively.

For measurements of Q̇, the stroke volume (SV) was first calculated as SV = VTI × CSA, where VTI is the measured velocity time integral of blood flow across the aortic valve and represents the distance the blood travels in the aortic outflow tract with each ventricular contraction, and CSA is the measured cross-sectional area of the aortic valve (in a parasternal long-axis view). From this, Q̇ was calculated as Q̇ = SV × HR, where HR is heart rate. Up to five measurements of Q̇ per minute were made during *Protocol 3*. For each volunteer, single values for Q̇ under conditions of euoxia (Q̇_euoxia_) and hypoxia (Q̇_hypoxia_) were calculated by averaging all measurements of Q̇ noted in the first 5 min of isocapnic euoxia and in the last 6 min of isocapnic hypoxia of *Protocol 3*, respectively.

#### Measurement and modeling of the acute hypoxic ventilatory response in Protocol 1.

Ventilatory volumes were measured using a bidirectional turbine volume-measuring device and flows (phase transitions) by a pneumotachograph; inspired and expired volumes were determined in real time by a computer. To quantify the ventilatory responses to acute variations in hypoxia, dynamic models relating total expired ventilation (V̇_E_) to end-tidal gas profiles were fitted to the data. Model 3 of Clement and Robbins (13) was employed. This is a single-compartment model in which the ventilatory sensitivity to acute hypoxia is modeled using a parameter G_P_ and a parameter V̇_C_ reflects residual V̇_E_ in the absence of hypoxia. The model was fit for each individual experiment in conjunction with a stochastic model based on a Kalman filter to describe the correlation that is present between successive breaths (23). A time constant (τ), pure delay (D), G_P_, and V̇_C_ were estimated for each data set. All of the parameters were constrained to be positive and the time parameters <30 s. The parameter values were estimated by nonlinear regression (Fortran Library routine EO4FDF; Numerical Algorithms Group, Oxford, UK) to minimize the sum of squares of the residuals.

#### Plasma erythropoietin measurements.

Venous blood samples were collected in tubes coated with EDTA at 0, 4, and 8 h of sustained hypoxia. The plasma was separated by centrifugation and frozen at −80°C until analysis. Plasma erythropoietin levels were determined by a human erythropoietin enzyme-linked immunosorbent assay (Quantikine IVD; R&D Systems, Abingdon, UK).

#### Other blood investigations.

Full blood count, serum iron, ferritin, transferrin, and transferrin saturation were determined in a clinical laboratory using standard procedures.

#### Experimental procedure with PBL.

Seven Tibetan and seven Han Chinese volunteers participated in this component of the study. Forty milliliters of blood was collected from each volunteer and centrifuged over a density gradient medium (Ficoll-Paque Plus; Amersham Biosciences, Little Chalfont, UK) to isolate the mononuclear cells. These were then incubated at 37°C for 30 min over a hydrophilic membrane to induce cellular adhesion by the monocytes, leaving a supernatant containing the PBL. The PBL were aliquoted to gas-permeable culture dishes and cultured at eight different oxygen tensions (20%, 10%, 5%, 2%, 1%, 0.5%, 0.2%, and 0.1%) for 20 h as previously described (9). Total RNA was then harvested from the PBL after incubation (Tri-Reagent; Sigma Aldrich, Poole, UK) according to standard protocol. Reverse transcriptase quantitative PCR (RTqPCR) was used to determine the expression of four HIF-regulated mRNA transcripts: vascular endothelial growth factor A, adrenomedullin, aldolase C, and prolyl-4-hydroxylase-alpha1; and also of HIF2α and *EGLN1* mRNA transcripts, using TaqMan primers and probes (Applied Biosystems, Warrington, UK). β2-microglobulin, which is not induced by hypoxia (9) (38), was used as an endogenous reference gene. The lack of induction of β2-microglobulin by hypoxia was also confirmed in this study by finding that the cycle threshold (CT) for β2-microglobulin with RTqPCR did not significantly vary with oxygen concentration (data not shown). Expression (2^−ΔΔCT^) was assessed in each sample relative to a standard calibrator cDNA sample that was run on each qPCR plate. ΔΔCT values were then calculated for each sample, where ΔΔCT = (CT_target gene_ − CT_reference gene_)_experimental sample_ − (CT_target gene_ − CT_reference gene_)_calibrator sample_.

#### Genotyping.

DNA was extracted either from blood (PaxGene Blood DNA Kit; Qiagen) or from saliva (Oragene DNA collection kit; Genotek, Ottawa, Canada) from 28 Tibetan volunteers that included the 14 participants of the physiology study. Samples were genotyped using the Illumina Infinium Human 610-Quadv1 BeadChip and a principal component analysis was performed. Genotyping at additional single nucleotide polymorphisms (SNPs) in *EPAS1* and *EGLN1* was carried out by either TaqMan SNP genotyping assays (Applied Biosystems) or by Sanger sequencing of relevant DNA regions.

#### Statistical analysis.

To take into account repeated measures on the same subject, statistical analyses of the data were predominantly carried out by fitting linear mixed effects models using the nlme package in R (version R2.13.0; Vienna, Austria). A number of different models were employed appropriate to the particular analysis; details of these appear in the appendix. The usual diagnostic analyses were carried out on the model residuals to confirm the requirements of normality and stability of variance. Student's *t*-tests were also used where appropriate. Statistical significance was reported at *P* < 0.05.

## RESULTS

**Fig. 1.**
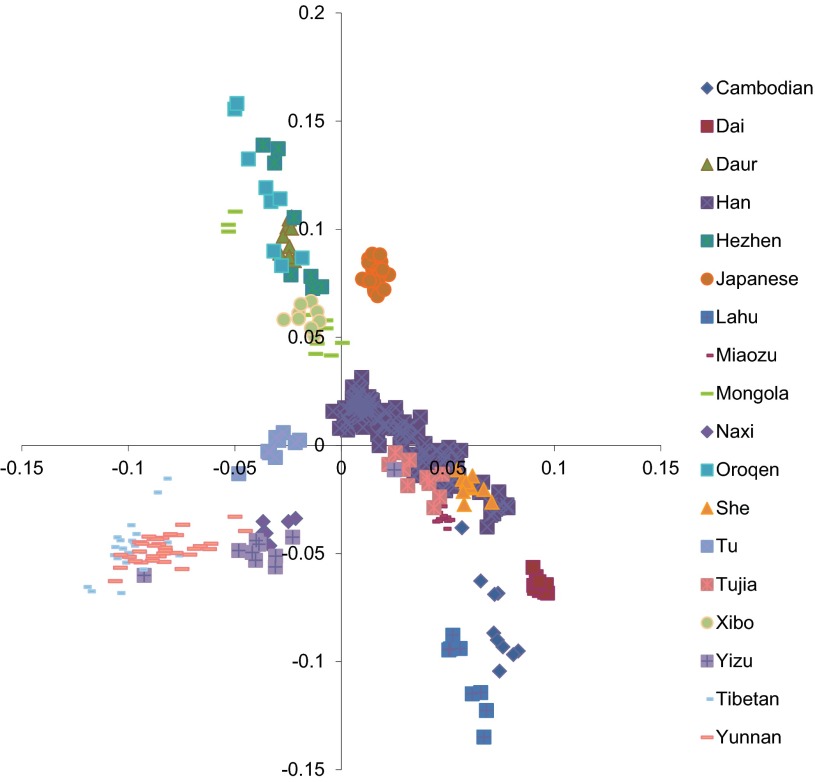
Principal component analysis among Asian populations. Data are from the Human Genome Diversity Project data set of Asian ethnicities, Hapmap, reference (5), and this study. The 28 Tibetan DNA samples from our study are represented by light blue, short horizontal lines and cluster together with the Yunnan Tibetan volunteers from reference (5).

A principal components analysis was undertaken on the data from the whole genome genotyping (Fig. 1). The results were compared with other Tibetan and Asian samples, and these provided genetic confirmation of the ancestry of the Tibetan volunteers in our study.

### 

#### Tibetans have significantly lower hemoglobin concentration and hematocrit but significantly higher resting ventilation (relative to metabolism) than Han Chinese at sea level.

Table 1 summarizes the baseline measurements. The hemoglobin concentration and hematocrit are significantly lower in Tibetans, whereas indices of iron balance are similar. Pet_CO_2__ is significantly lower in Tibetans, suggesting that they have a higher ratio of pulmonary ventilation to metabolism at rest than Han Chinese.

**Table 1.** Baseline characteristics and physiological parameters during air breathing

Parameter	Tibetan (*n* = 14)	Han Chinese (*n* = 13)	*P* (unpaired *t*-test)
Age, years	30.9 ± 5.5	27.6 ± 5.4	NS
Height, m	1.73 ± 0.05	1.74 ± 0.04	NS
Weight, kg	73.8 ± 9.1	70.5 ± 8.2	NS
Hemoglobin, g/dl	14.2 ± 0.9	15.3 ± 1.2	< **0.05**
Hematocrit, l/l	0.43 ± 0.03	0.47 ± 0.04	< **0.05**
Ferritin, μg/l	214 ± 99	168 ± 86	NS
Transferrin saturation, %	35 ± 9	34 ± 8	NS
End-tidal Pco_2_, mmHg	37.8 ± 2.4	40.7 ± 2.5	< **0.005**

Values are mean ± SD. NS, not significant.

#### Tibetans living at sea level have a blunted pulmonary vascular response to acute and sustained hypoxia, and show a different pattern of early pulmonary vascular acclimatization to sustained hypoxia than Han Chinese lowlanders.

**Fig. 2.**
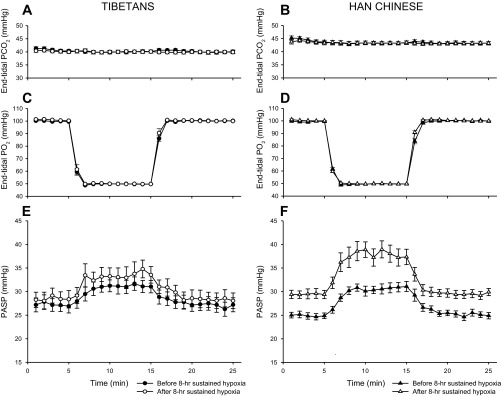
Pulmonary vascular response to acute isocapnic hypoxia before and after 8 h of sustained isocapnic hypoxia. *A* and *B*: end-tidal partial pressure of CO_2_ (Pet_CO_2__) against time for Tibetan and Han Chinese, respectively, during the acute hypoxic protocol. *C* and *D*: end-tidal partial pressure of O_2_ (Pet_O_2__) against time for Tibetan and Han Chinese, respectively. *E* and *F*: pulmonary arterial systolic pressure (PASP) values against time for 10 Tibetan and 10 Han Chinese volunteers, respectively. Values are means; error bars represent SEM.

Figure 2 shows the mean values for PASP every minute during acute exposures to hypoxia for Tibetan and Han Chinese volunteers. The within-subject standard deviation (SD) for individual determinations of PASP was 1.2 mmHg. For both groups, responses to acute hypoxia were measured before and after an 8-h exposure to sustained isocapnic hypoxia. Mean values for the sensitivity of PASP to acute change in hypoxia (G_PASP_) together with mean values for PASP during conditions of euoxia and hypoxia are reported in Table 2. Tibetans have significantly lower values for G_PASP_ than Han Chinese (*P* < 0.05). Tibetans and Han Chinese did not differ in their initial values for PASP under conditions of euoxia. After conditioning with an 8-h exposure to sustained hypoxia, there was an increase in both G_PASP_ and in baseline (euoxic) PASP in both Tibetans and Han Chinese. However, the rise and persistent elevation of the baseline PASP, itself a feature of early pulmonary vascular acclimatization to sustained hypoxia (14), was significantly lower in Tibetans than in Han Chinese (*P* < 0.0002), as illustrated in Fig. 2.

**Table 2.** Physiological parameters before and after 8 h of sustained isocapnic hypoxia

	Tibetan (*n* = 14)		Han Chinese (*n* = 13)	
Parameter	Prior to 8-h sustained hypoxia	Post 8-h sustained hypoxia	Prior to 8-h sustained hypoxia	Post 8-h sustained hypoxia
V̇_C_, l/min	10.7 ± 2.4	19.7 ± 4.9	12.6 ± 3.0	18.9 ± 9.1
G_P_, l·min^−1^·%^−1^	0.36 ± 0.14	0.98 ± 0.58	0.50 ± 0.46	0.97 ± 0.92
PASP_euoxia_, mmHg	26.8 ± 3.9	28.2 ± 4.9	24.8 ± 1.7	29.5 ± 2.7
PASP_hypoxia_, mmHg	30.8 ± 3.5	33.7 ± 5.3	30.5 ± 2.7	37.6 ± 5.1
ΔSaO_2_, %	12.9 ± 0.2	12.9 ± 0.2	12.9 ± 0.1	13.0 ± 0.2
G_PASP_, mmHg/%	0.32 ± 0.12	0.43 ± 0.16	0.45 ± 0.11	0.62 ± 0.20
Q̇_euoxia_, l/min	4.8 ± 0.5	4.9 ± 0.5	4.8 ± 0.5	5.2 ± 0.7
Q̇_hypoxia_, l/min	5.5 ± 0.7	6.0 ± 0.7	5.8 ± 0.6	6.2 ± 0.6
G_Q̇_, l·min^−1^·%^−1^	59 ± 25	82 ± 40	77 ± 18	79 ± 20
Plasma erythropoietin, miU/ml	8.5 ± 3.7	16.1 ± 8.2	8.9 ± 3.2	17.2 ± 9.3

Values are mean ± SD. V̇_C_, residual ventilation in the absence of hypoxia (in isocapnia). G_P_, acute hypoxic ventilatory sensitivity. PASP_euoxia_ and PASP_hypoxia_ are pulmonary arterial systolic pressures under conditions of euoxia and hypoxia, respectively. Q̇_euoxia_ and Q̇_hypoxia_ are cardiac output under conditions of euoxia and hypoxia, respectively. G_PASP_, the sensitivity of the pulmonary vascular response to hypoxia, is calculated as G_PASP_ = (PASP_hypoxia_ − PASP_euoxia_)/ΔSaO_2_, where ΔSaO_2_ is the percentage drop in hemoglobin oxygen saturation upon exposure to acute isocapnic hypoxia. Similarly, G_Q̇_, the sensitivity of the cardiac output response to hypoxia, is calculated as Ġ_Q_ = (Q̇_hypoxia_ − Q̇_euoxia_)/ΔSaO_2_. Statistical analyses of the data were carried out by fitting linear mixed-effects models. Note that 2 out of 14 Tibetan volunteers did not have tricuspid regurgitation and thus do not contribute to any PASP parameters. A further 2 out of 14 did not complete the acute hypoxic protocols following the 8 h of sustained hypoxia, and hence do not contribute to the *Post 8-h isocapnic hypoxia* column, except for plasma erythropoietin. Two out of 13 Han Chinese volunteers did not complete the 8-h sustained hypoxia exposure and hence do not contribute to the *Post 8-h isocapnic hypoxia* column. A further 1 out of 13 did not complete the acute hypoxic protocols (*Protocols 2* and *3*) following the 8-h exposure to sustained hypoxia and hence does not contribute to any PASP or Q̇ parameters in the *Post 8-h isocapnic hypoxia* column.

**Fig. 3.**
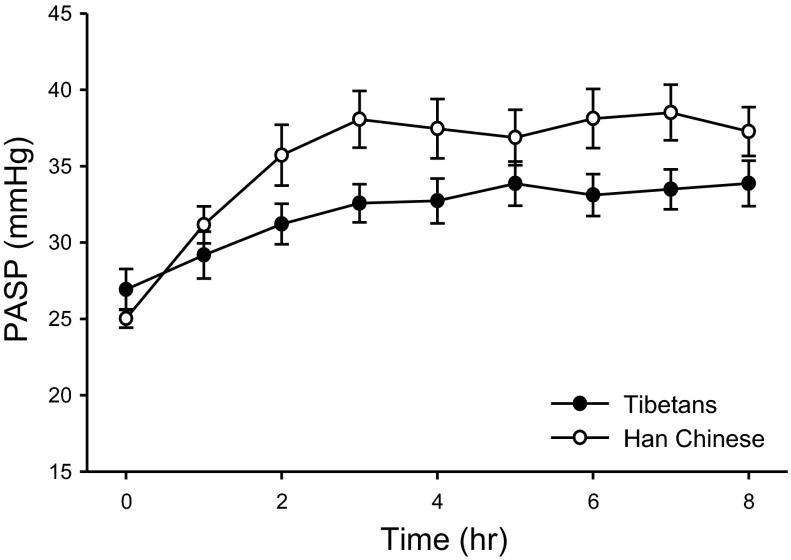
Pulmonary vascular response during 8 h of sustained isocapnic hypoxia. Closed circles represent PASP results from 10 Tibetan volunteers; open circles are results from 10 Han Chinese volunteers. Values are means; error bars indicate SEM.

Figure 3 shows the mean PASP measured at hourly intervals for Tibetan and Han Chinese volunteers during the 8-h exposure to sustained isocapnic hypoxia. The time-dependent rise in PASP in response to sustained hypoxia is significantly lower in Tibetans than in Han Chinese (*P* < 0.0001).

**Fig. 4.**
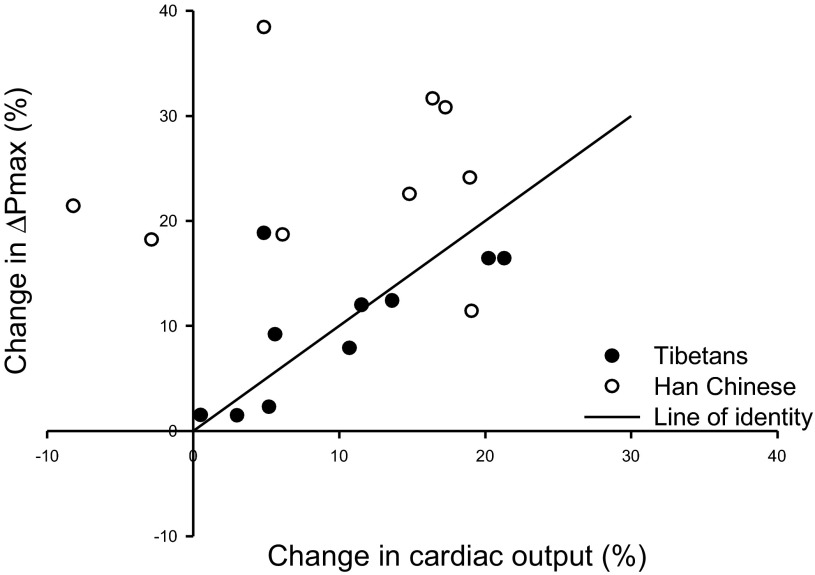
Percentage change in ΔPmax against percentage change in cardiac output after 8 h of hypoxia for each individual. Closed circles represent data from Tibetan and open circles from Han Chinese volunteers.

To explore the relationship between the pressure across the pulmonary circulation and cardiac output, the percentage increase in ΔPmax was plotted against the percentage increase in cardiac output (Q̇) after 8 h of hypoxia, as shown in Fig. 4. We found that *1*) the data for the Han Chinese lie significantly above the line of identity (*P* < 0.01), showing that the percentage increase in pressure across the pulmonary circulation is significantly greater than the percentage increase in cardiac output; *2*) the data for the Tibetans do not depart significantly from the line of identity; and *3*) the responses of the Han Chinese are significantly different from those of the Tibetans (*P* < 0.005).

#### Tibetans and Han Chinese do not differ in their acute hypoxic ventilatory sensitivity (G_P_) at sea level and exhibit similar early ventilatory acclimatization to sustained isocapnic hypoxia.

**Fig. 5.**
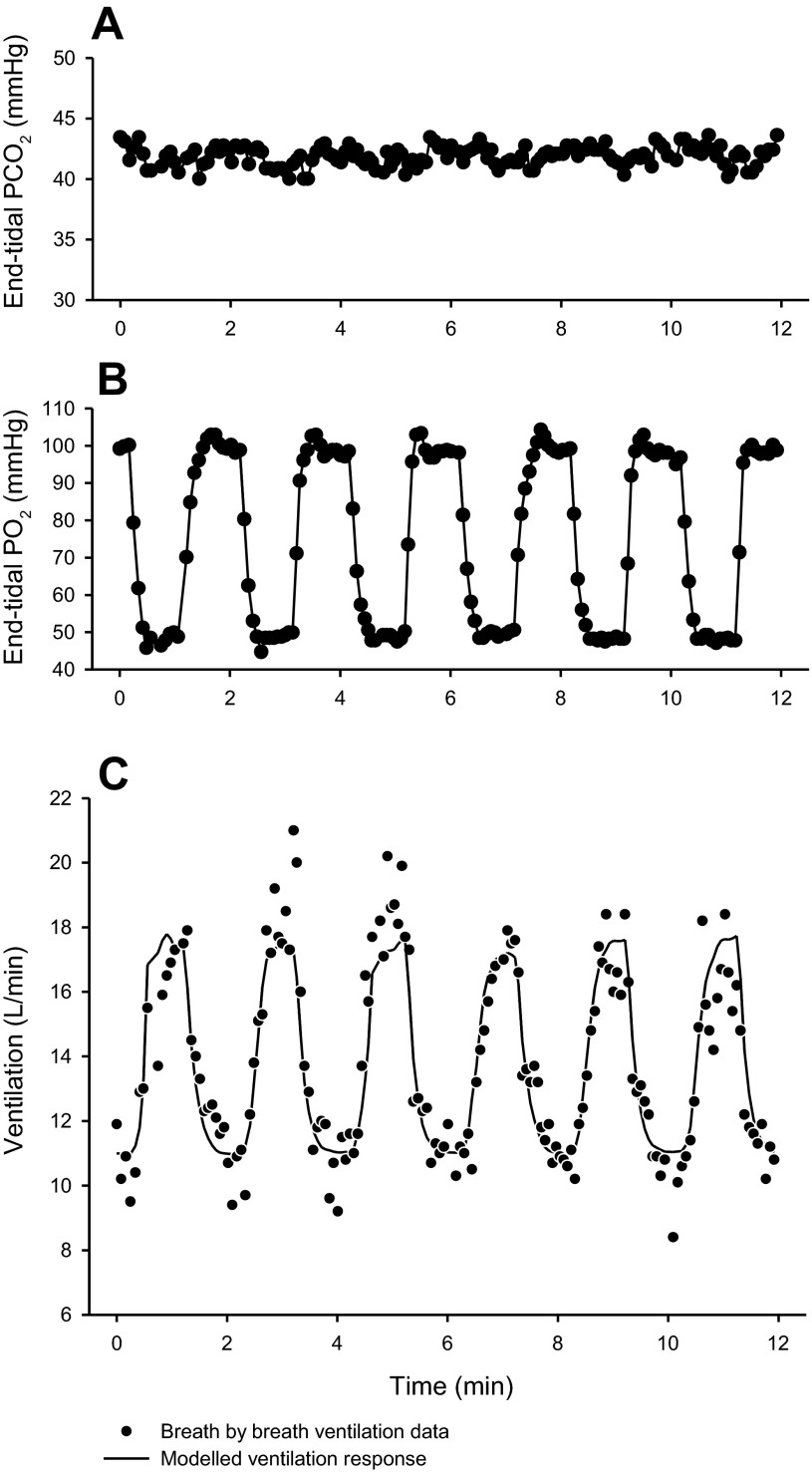
Example of a volunteer's ventilatory response during the acute cyclical hypoxic challenge of *Protocol 1*. *A* and *B*: end-tidal partial pressure of CO_2_ and O_2_, respectively, against time. *C*: corresponding ventilation measurements (breath by breath) against time.

A first-order model was used to fit the ventilatory response to acute hypoxia both before and after an 8-h conditioning exposure to sustained isocapnic hypoxia (see Fig. 5 as an example). Mean values for the ventilatory sensitivity to acute hypoxia (G_P_) and the residual ventilation in the absence of hypoxia (V̇_C_) are reported in Table 2. These did not differ significantly between Tibetans and Han Chinese. In both Tibetans and Han Chinese, G_P_ significantly increased following 8 h of sustained hypoxia (*P* < 0.001)—a feature of early ventilatory acclimatization to sustained hypoxia—but the magnitude of this increase did not depend on ethnicity.

#### Tibetans and Han Chinese do not significantly differ in the response of their cardiac output to hypoxia nor do they differ in their erythropoietic response to sustained hypoxia.

Mean values for cardiac output indices and plasma erythropoietin are reported in Table 2. The within-subject SD for individual determinations of cardiac output was 0.3 L/min. No statistically significant differences were found.

#### HIF2α mRNA but not EGLN1 mRNA levels are significantly lower in PBL from Tibetan than from Han Chinese volunteers.

**Fig. 6.**
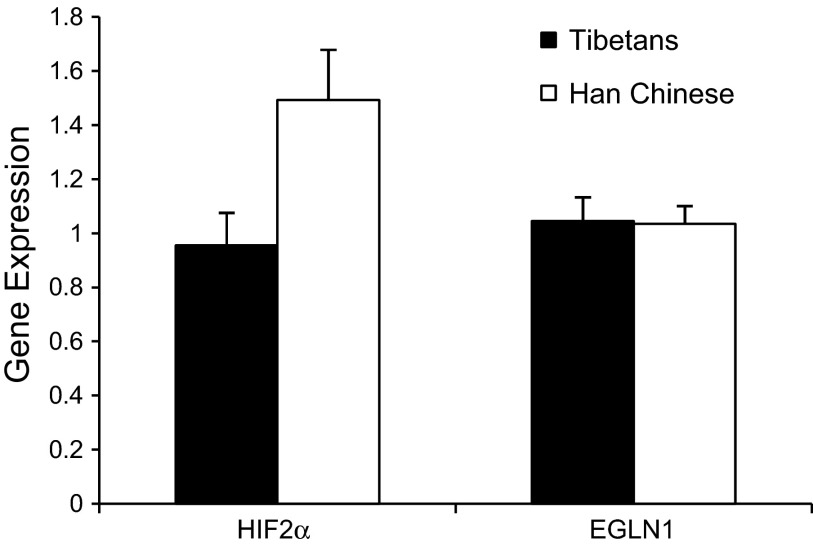
Expression of HIF2α mRNA and EGLN1 mRNA (relative to a standard calibrator sample) in PBL from seven Tibetan (filled bars) and seven Han Chinese (open bars) volunteers. Values are means; error bars are SEM.

Figure 6 shows the mean expression level of HIF2α and *EGLN1* mRNA in PBL from Tibetan and Han Chinese volunteers. The expression of HIF2α mRNA is significantly lower in PBL from Tibetans than Han Chinese (*P* < 0.05), whereas the expression of *EGLN1* mRNA is not significantly different between the two groups.

#### The induction of four HIF-target genes by hypoxia is significantly lower in PBL from Tibetan than from Han Chinese volunteers.

**Fig. 7.**
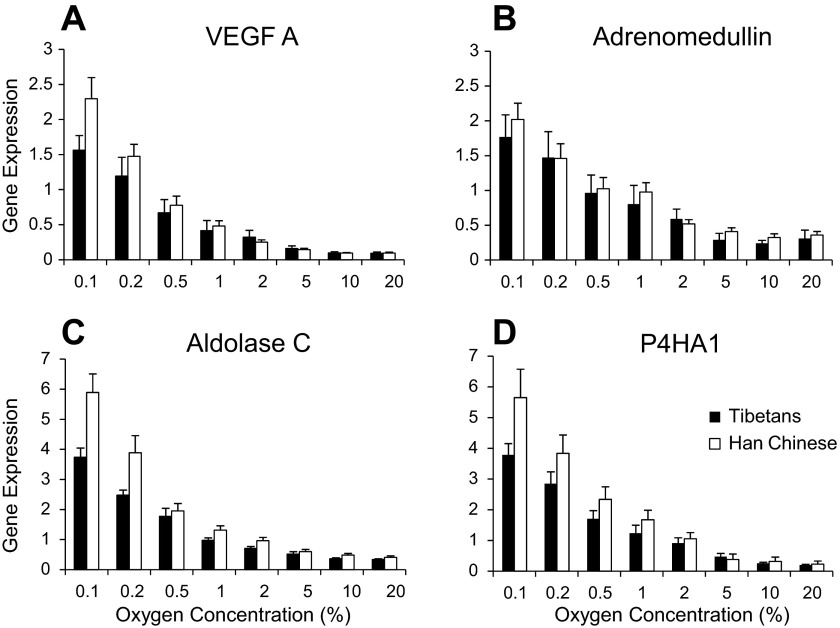
Expression of HIF-target genes (relative to a standard calibrator sample) at graded oxygen tension for seven Tibetan and seven Han Chinese volunteers. *A*: vascular endothelial growth factor A (VEGFA); *B*: adrenomedullin; *C*: aldolase C; *D*: prolyl-4-hydroxylase-alpha-1 (P4HA1). Filled bars represent results from Tibetan volunteers; open bars from Han Chinese volunteers. Values are means; error bars are SEM.

Figure 7 shows the expression of four HIF-target gene transcripts in PBL at eight different oxygen concentrations for Tibetan and Han Chinese volunteers. It was found that both the relative expression and the hypoxic induction of HIF-target genes are significantly lower in Tibetans compared with Han Chinese (*P* < 0.05 and *P* < 0.05, respectively).

#### The rise in plasma erythropoietin with time in sustained hypoxia in Tibetans is significantly associated with genotype at both the EPAS1 and the EGLN1 loci.

As the particular causative mutation under selection at either the *EPAS1* or the *EGLN1* locus has yet to be identified, an appropriate marker SNP has to be selected for each gene to assign the genotype to each individual. This section describes the assignment of genotype and the associations.

**Fig. 8.**
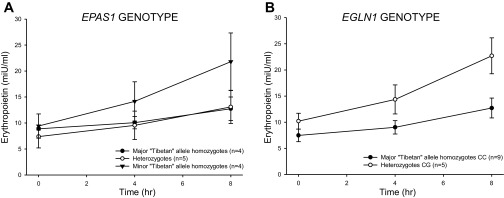
Plasma erythropoietin against time in sustained hypoxia in the chamber for Tibetan volunteers according to *EPAS1* genotype (*A*) and *EGLN1* genotype (*B*). Values are means; error bars are SEM.

Of the eight genome-wide significant SNPs defining the signal of selection at *EPAS1* in the study by Beall et al. (5), rs1868092 was the SNP that correlated most significantly with hemoglobin. It was also in 100% linkage disequilibrium with rs13006131, which correlated with hemoglobin in an independent sample (in which rs1868092 was not genotyped). Using rs1868092 as a marker for the selected haplotype at *EPAS1*, Fig. 8*A* shows the plasma erythropoietin against time in sustained hypoxia for the three *EPAS1* genotypes in the Tibetan volunteers (allele A is the major allele selected for within the Tibetan population). The rise in plasma erythropoietin with 8 h of sustained hypoxia is significantly higher in the minor homozygous (GG) compared with the major homozygous (AA) Tibetan volunteers (*P* < 0.05). Table 3 shows the results by *EPAS1* genotype for other physiological parameters. These exhibit a general (but not statistically significant) tendency for the “Tibetan” allele to be associated with smaller responses to hypoxia.

**Table 3.** Physiological parameters before and after 8 h of sustained isocapnic hypoxia for Tibetan volunteers according to EPAS1 genotype

	Major “Tibetan” allele homozygotes (*n* = 4)		Heterozygotes (*n* = 5)		Minor “Tibetan” allele homozygotes (*n* = 4)	
Parameter	Prior to 8-h sustained hypoxia	Post 8-h sustained hypoxia	Prior to 8-h sustained hypoxia	Post 8-h sustained hypoxia	Prior to 8-h sustained hypoxia	Post 8-h sustained hypoxia
Hb, g/dl	13.9 ± 0.4		14.1 ± 0.5		14.7 ± 1.6	
Hematocrit, l/l	0.42 ± 0.01		0.44 ± 0.04		0.44 ± 0.05	
Pet_co_2__, mmHg	37.8 ± 2.7		37.7 ± 3.0		37.9 ± 1.4	
V̇_C_, L/min	9.2 ± 0.7	16.3 ± 3.5	10.7 ± 1.7	20.0 ± 1.6	12.1 ± 3.7	22.8 ± 6.5
G_P_, l·min^−1^·%^−1^	0.30 ± 0.10	0.54 ± 0.31	0.34 ± 0.12	1.27 ± 0.46	0.45 ± 0.19	1.14 ± 0.71
PASP_euoxia_, mmHg	26.1 ± 4.3	26.9 ± 3.9	27.2 ± 2.7		26.9 ± 5.5	28.7 ± 6.7
PASP_hypoxia_, mmHg	29.9 ± 4.0	31.7 ± 4.5	30.7 ± 2.0		31.9 ± 4.8	35.3 ± 6.3
G_PASP_, mmHg/%	0.30 ± 0.12	0.37 ± 0.17	0.27 ± 0.11		0.4 ± 0.1	0.5 ± 0.2
Q̇_euoxia_, l/min	4.6 ± 0.4	4.7 ± 0.5	4.8 ± 0.5	5.2 ± 0.1	4.8 ± 0.6	5.0 ± 0.6
Q̇_hypoxia_, l/min	5.4 ± 0.6	5.8 ± 0.5	5.5 ± 0.8	5.7 ± 0.5	5.7 ± 1.0	6.4 ± 0.8
G_Q̇_, ml·min^−1^·%^−1^	57 ± 22	84 ± 34	52 ± 24	38 ± 30	70 ± 31	112 ± 23
Plasma erythropoietin, miU/ml	8.9 ± 0.5	12.7 ± 3.9	7.4 ± 4.9	13.1 ± 7.1	9.4 ± 4.7	21.8 ± 11.0

Values are mean ± SD. Major allele refers to the allele that is selected for within the Tibetan population. All the major homozygous and minor homozygous volunteers participated in all protocols of the physiology study and feature in all columns. Because four out of five heterozygous volunteers either did not have tricuspid regurgitation on echocardiography or did not complete the acute hypoxic protocols following 8 h of sustained hypoxia the boxes relating to PASP in the *Post 8-h hypoxia* column are left blank (as data from only one heterozygous volunteer were available) to avoid confusion. One individual's genotype could not be classified and was hence excluded from the analysis.

At the *EGLN1* locus, the Tibetan DNA samples were genotyped for two *EGLN1* noncoding SNPs, rs2275279 and rs961154, which have allelic frequencies that differ markedly between Tibetan and Han Chinese (29, 36), and which formed part of a positively selected *EGLN1* haplotype that correlated with hemoglobin in (36). We also genotyped the Tibetan samples for two coding variants in exon 1, rs12097901 (380G>C, cysteine to serine substitution; C127S) and rs186996510 (12C>G, aspartate to glutamate substitution; D4E), which have been previously observed at high frequencies in Tibetans (24), a finding confirmed here (Table 4). We assessed the degree of linkage disequilibrium between these four variants. Rs12097901 (C127S) was highly correlated with both rs2275279 and rs961154, which comprised the *EGLN1* selected haplotype in (36), but rs186996510 was not, as shown in Table 5. Using rs12097901 as a marker for the selected haplotype, Fig. 8*B* shows the plasma erythropoietin plotted against time in sustained hypoxia for Tibetan volunteers by genotype (C is the major allele for this in Tibetans). The rise in plasma erythropoietin with time in sustained hypoxia is significantly higher in the heterozygous (CG) than in the homozygous (CC) Tibetan volunteers (*P* < 0.01); there were no homozygous (GG) volunteers. Table 6 shows the results by genotype for other physiological parameters.

**Table 4.** Allelic frequencies for the two coding variants in EGLN1 in the Tibetan cohort (n = 28) genotyped in this study

Coding variant	Frequency of allele 1	Frequency of allele 2
rs12097901 (380G>C, C127S)	0.81 (C)	0.19 (G)
rs186996510 (12C>G, D4E)	0.65 (G)	0.35 (C)

**Table 5.** Correlation coefficients between the EGLN1 SNPs

	rs12097901 (C127S)	rs186996510 (D4E)	rs961154	rs2275279
rs12097901 (C127S)		*r*^2^ = 0.613 (*P* = 0.001)	*r*^2^ = 0.619 (*P* = 0.002)	*r*^2^ = 0.625 (*P* = 0.002)
rs186996510 (D4E)			*r*^2^ = 0.215 (*P* = 0.264)	*r*^2^ = 0.428 (*P* = 0.03)
rs961154				*r*^2^ = 0.816 (*P* = 0.000)
rs2275279				

**Table 6.** Physiological parameters before and after 8 h of sustained isocapnic hypoxia for Tibetan volunteers according to EGLN1 genotype

	Major “Tibetan” allele homozygotes (CC) (*n* = 9)		Heterozygotes (CG) (*n* = 5)	
Parameter	Prior to 8-h sustained hypoxia	Post 8-h sustained hypoxia	Prior to 8-h sustained hypoxia	Post 8-h sustained hypoxia
Hb, g/dl	14.5 ± 1.1		13.80 ± 0.34	
Hematocrit, l/l	0.44 ± 0.04		0.42 ± 0.01	
Pet_co_2__, mmHg	38.2 ± 2.5		36.9 ± 1.9	
V̇_C_, l/min	10.4 ± 2.5	19.3 ± 5.0	11.0 ± 2.5	20.2 ± 5.1
G_P_, l·min^−1^·%^−1^	0.38 ± 0.16	1.07 ± 0.69	0.33 ± 0.12	0.86 ± 0.42
PASP_euoxia_, mmHg	26.3 ± 4.8	28.3 ± 7.1	27.4 ± 2.5	28.1 ± 2.1
PASP_hypoxia_, mmHg	29.9 ± 4.4	33.1 ± 7.4	32.1 ± 1.6	34.3 ± 2.6
G_PASP_, mmHg/%	0.28 ± 0.08	0.37 ± 0.05	0.36 ± 0.16	0.48 ± 0.21
Q̇_euoxia_, l/min	4.7 ± 0.4	4.7 ± 0.5	4.8 ± 0.6	5.0 ± 0.5
Q̇_hypoxia_, l/min	5.4 ± 0.6	5.8 ± 3.8	5.6 ± 1.0	6.1 ± 1.1
G_Q̇_, ml·min^−1^·%^−1^	53 ± 18	83 ± 34	66 ± 35	88 ± 50
Plasma erythropoietin, miU/ml	7.5 ± 3.6	12.7 ± 5.7	10.2 ± 3.4	23.7 ± 7.5

Values are mean ± SD. The C allele refers to the allele resulting in cysteine to serine substitution (C127S) and is found at high frequencies in Tibetans. Two out of 14 volunteers (both homozygous) did not have tricuspid regurgitation and thus do not contribute to any PASP parameters. A further 2 out of 14 (both homozygous) did not complete *Protocols 1, 2*, and *3* following the 8-h exposure to sustained hypoxia, and hence do not contribute to the *Post 8-h isocapnic hypoxia column*, except for plasma erythropoietin.

## DISCUSSION

This study demonstrates that Tibetan natives living at sea level exhibit a significantly different integrative-physiology phenotype compared with Han Chinese lowlanders. The differences are observed across a range of physiological variables of relevance to oxygen homeostasis. The differences relate both to basal measurements made during air-breathing conditions and measurements in response to hypoxic challenges and encompass erythropoietic, pulmonary vascular, and ventilatory regulation.

We found that even at sea level Tibetans had a hemoglobin concentration that was around 1 g/dl lower than Han Chinese. Two things are of note. First, this difference appears to be smaller than the difference observed between Tibetans and other groups at high altitude, where Tibetan highlanders have hemoglobin concentrations typically 1 to 3.5 g/dl lower than their Andean counterparts or Han Chinese migrants to high altitude (4, 17, 27, 42). This suggests that the differences between Tibetans and Han Chinese relate both to their set point for hemoglobin concentration in the absence of environmental hypoxia, and the induction of hemoglobin by reductions in ambient Po_2_. Second, the difference in hemoglobin concentration between Tibetan and Han Chinese comfortably exceeds the maximum difference observed in a number of genome-wide studies exploring genetic determinants of hemoglobin concentration at sea level (7, 11, 39); the largest effect observed to date was of 0.13 g/dl per copy of the variant rs855791 for TMPRSS6 (11).

The most striking difference that was observed between Tibetans and Han Chinese was the difference in their pulmonary vascular responses to hypoxia. Our study is the first to assess pulmonary arterial vascular responses to hypoxia in Tibetans living at sea level and to demonstrate the marked reduction in sensitivity to both acute and subacute (8-h) hypoxia. Analogous experiments have not been performed in Andeans, although Andeans exhibit at least some reversal of pulmonary hypertension after migrating to live at sea level for 2 or more years (35). However, our results are consistent with a previous study of five Tibetans resident at 3,658 m that found that their pulmonary arterial pressures were within sea-level norms and were little changed by additional exposure to further hypoxia (18).

Figure 4 explores the relationship between the changes in cardiac output and those in pulmonary arterial pressure in response to the 8-h exposure to hypoxia. We show that the differences between Tibetans and Han Chinese in their pulmonary vascular responses to hypoxia are not attributed to differences in cardiac output. For the Han Chinese, the percentage increments in pulmonary pressure are significantly greater than the percentage increments in cardiac output, demonstrating that the rise in pulmonary pressure cannot simply be the result of increased flow across a fixed pulmonary vascular resistance. For the Tibetans, this was not the case. This could either be explained by the increment in cardiac output generating the increment in pulmonary pressure or, alternatively, it could be explained by interindividual differences in response to 8 h of hypoxia, which affects the systemic and pulmonary vasculature in a similar manner. Of these two explanations the latter appears to be consistent with the data of Balanos et al. (3) who showed that within an individual the pulmonary arterial pressure varied little with natural variations of cardiac output both in conditions of euoxia and hypoxia, quoting a value of 0.6 mmHg per liter per minute, and suggested that less than 10% of the rise in pulmonary artery pressure produced by hypoxia could be caused by the concomitant increase in cardiac output.

Hypoxic pulmonary vasoconstriction can be a beneficial mechanism at sea level where it helps to maintain ventilation-perfusion matching within the lung. However, the environmental hypoxia of high altitude causes global pulmonary vasoconstriction, which leads to pulmonary hypertension and an increase in right ventricular work, all of which is likely to be maladaptive. Indeed, hypoxia-induced pulmonary hypertension characterizes chronic mountain sickness, a disease with high mortality and morbidity in high-altitude populations, and quite prevalent in Andeans, but of low prevalence in Tibetans (26). Thus, it may well be that the phenotype of a blunted hypoxic pulmonary vascular response is advantageous for survival at high altitude and could have been naturally selected by genetic adaptation in Tibetans. Nevertheless, it is also perfectly possible that there is some other phenotypic consequence of HIF hyporesponsiveness that is the primary phenotype under selection.

Studies of genetic selection investigating high-altitude adaptation have repeatedly identified *EPAS1* and *EGLN1* as loci that have undergone recent positive selection in Tibetans (5, 8, 29, 36, 43, 44) and in some of these studies, the putatively selected “Tibetan” genetic variants were associated with lower hemoglobin concentrations at high altitude (5, 36, 44). Despite this, we did not identify any difference in hemoglobin concentration when comparing Tibetans of different genotypes. Since it was possible to recruit only a limited number of Tibetans living at sea level in the UK, this could either be the result of a type II statistical error arising from a small sample size or it could arise because measurements were made at sea level where the effects of variation within the HIF pathway may be less apparent than in hypoxic environments. In keeping with the latter possibility, we did identify a statistically significant difference in erythropoietin regulation between genotype in Tibetans for both *EPAS1* and *EGLN1* during exposure of these individuals to 8 h of hypoxia. For both genes, the smaller erythropoietin response to sustained hypoxia was associated with the “Tibetan” variant.

One attractive hypothesis is that the relative insensitivity of the pulmonary vasculature to hypoxia in Tibetans arises through the genetic variation within the HIF system, which has been previously identified in studies of genetic selection (5, 36). In support of this, patients with disease caused by mutations in the HIF system exhibit both pulmonary hypertension and increased sensitivity of the pulmonary vasculature to hypoxia (15, 38). Furthermore, mice with heterozygous loss of HIF2α are resistant to development of hypoxic pulmonary hypertension (10). In our present study, even though the pulmonary vascular responses to hypoxia are noticeably different in Tibetans of different genotype in absolute physiological terms (Table 3 and Table 6), these differences fell short of statistical significance. This may be the result of a type II statistical error arising from the small number of Tibetans studied. Indeed, in the case of *EPAS1* but not *EGLN1*, there is evidence of a general tendency such that the “Tibetan” allele appears to be associated with smaller hypoxic responses across most physiological parameters tested (Table 3).

A potential complicating factor in our study is that all Tibetans spent the first 6 or more years of their life at high altitude before migrating to live at sea level. For this reason, it cannot be concluded with complete certainty that the differences between Tibetan and Han Chinese volunteers arise from genetic rather than environmental factors during growth and development. However, for Pet_CO_2__, which reflects the set point for the ratio of pulmonary ventilation to metabolism, there is evidence from South American populations that high-altitude residence during growth and development does not affect subsequent values for Pet_CO_2__ determined at sea level. In a study of ∼200 individuals the average difference in Pet_CO_2__ between high-altitude natives that had emigrated to live at sea level and sea-level natives was 0.1 mmHg (−0.6 to 0.8, 95% CI) (22). In the present study, the difference in Pet_CO_2__ between Tibetan and Han Chinese sea-level residents was 2.9 mmHg (1.0 to 4.9, 95% CI). In contrast with the results for Pet_CO_2__, Tibetans and Han Chinese did not differ in their baseline hypoxic ventilatory sensitivity or in early ventilatory acclimatization. This finding is in keeping with studies in Andean high-altitude natives at sea level who were found to have similar acute ventilatory responses to short-duration acute hypoxic stimuli and who upon reexposure to high-altitude hypoxia were also found to undergo ventilatory acclimatization in the same manner as sea-level natives (16, 32, 40). A further point to discuss is that all the volunteers in our study were men; there is a possibility that responses in women may differ, particularly in the control of breathing (21, 41).

Finally, to determine whether we could detect functional differences between Tibetans and Han Chinese in the HIF system at the cellular level, we also examined expression of HIF-regulated genes in PBL. We found that both the relative expression and the hypoxic induction of HIF-regulated genes were significantly lower in PBL from Tibetan volunteers, consistent with a reduced sensitivity of the HIF response in Tibetans. This finding parallels the observations of reduced hypoxic sensitivities in Tibetans at the integrative physiology level and further supports the importance of variation in the HIF hydroxylase system in these effects.

The advantage of using PBL in the cellular experiments is that they represent primary cells that are easily and noninvasively obtainable from circulating blood and have been found to exhibit hypoxic regulation in a large number of genes. A limitation is that the expression profiles of genes can vary significantly in a tissue-specific manner and so differences (or absence of such) in expression of some genes in lymphocytes may or may not be found in other cell types. For *EGLN1*, we found no differences in expression at the mRNA level between Tibetans and Han Chinese. Although this finding is limited to PBL and may be different in other cell types, it could suggest that any difference between Tibetan and Han Chinese arises from differences at the protein level. In support of this, two coding variants have been described with particularly high frequency in Tibetans (24), although the functional significance of this is not yet known. These coding variants are nonsynonymous, resulting in amino acid substitutions that may affect protein stability, trafficking, or enzymatic activity. For *EPAS1*, no coding genetic variation has been identified thus far through sequencing in Tibetans, and all genome-wide significant or hemoglobin-associating variants described are in noncoding regions. Here, however, we found significantly reduced HIF2α mRNA in PBL from Tibetans compared with Han Chinese. In this case, it seems more likely that functional variation in *EPAS1* arises through effects on transcription of *EPAS1*. Further functional molecular studies are required to investigate these possibilities.

In summary, this study provides further evidence that Tibetans respond less vigorously to hypoxic challenge. The study also demonstrates that this is the case at sea level as well as at high altitude, and it extends the evidence that the physiological differences in Tibetans arise, at least in part, due to a hyporesponsive HIF transcriptional system.

## GRANTS

Support for this study was provided by a Wellcome Trust Clinical Training Research Fellowship Grant 089457/Z/09/Z to N. Petousi.

## DISCLOSURES

No conflicts of interest, financial or otherwise, are declared by the author(s).

## AUTHOR CONTRIBUTIONS

Author contributions: N.P., P.J.R., and P.A.R. conception and design of research; N.P., Q.P.P.C., G.L.C., H.-Y.C., F.F., K.I., M.M., K.V.S., and N.P.T. performed experiments; N.P., D.L., and P.A.R. analyzed data; N.P., D.L., P.J.R., and P.A.R. interpreted results of experiments; N.P., G.L.C., and M.M. prepared figures; N.P., P.J.R., and P.A.R. drafted manuscript; N.P., G.L.C., P.J.R., and P.A.R. edited and revised manuscript; N.P., Q.P.P.C., G.L.C., H.-Y.C., F.F., K.I., D.L., M.M., K.V.S., N.P.T., P.J.R., and P.A.R. approved final version of manuscript.

## Footnotes

1 This article is the topic of an Invited Editorial by Simonson and Powell (37a).

## Appendix

For analyses examining the influence of ethnicity on the physiological responses to acute hypoxia, the following linear mixed-effects model was used:

$${\text{y}}_{\text{i}}=ã\;+\beta {\text{x}}_{\text{i}}+\gamma {\text{z}}_{\text{i}}+\delta {\left(\text{x}\times \text{z}\right)}_{\text{i}}+\varepsilon_{\text{i}}$$

where for the ith observation, x_i_ is a binary fixed effect representing ethnicity (i.e., being Tibetan or Han Chinese) and z_i_ is a binary fixed effect representing before or after 8 h of sustained hypoxia. ã ∼ N (α, σ_α_^2^) is a random variable reflecting intervolunteer variability and ε_i_ ∼ N(0, σ^2^) is the interobservation error.

For the statistical analysis examining the influence of ethnicity on the time-dependent rise of PASP during sustained hypoxia the following linear mixed-effects model was used:

$${\text{y}}_{\text{i}}=ã\;+\beta {\text{x}}_{\text{i}}+\sum_{\text{k}=1}^{8}\gamma_{\text{k}}{\text{z}}_{{\text{k}}_{\text{i}}}+\sum_{\text{k}=1}^{8}\delta_{\text{k}}{\left(\text{x}\times {\text{z}}_{\text{k}}\right)}_{\text{i}}+\varepsilon_{\text{i}}$$

where for the ith observation, x_i_ represents ethnicity and is a binary fixed effect, and z_k_i__ is a categorical fixed effect representing the kth hourly time point in sustained hypoxia over the 8-h period, ã ∼ N (α, σ_α_^2^) is a random variable reflecting intervolunteer variability, and ε_i_ ∼ N(0, σ^2^) is the interobservation error.

For the statistical analysis of the influence of genotype on the induction of plasma erythropoietin with time in sustained hypoxia in Tibetans, the following linear mixed-effect model was used for genotype at *EGLN1*:

$${\text{y}}_{\text{i}}=ã\;+\beta {\text{x}}_{\text{i}}+\overset{2}{\underset{\text{k}=1}{\sum }}\gamma_{\text{k}}{\text{z}}_{{\text{k}}_{\text{i}}}+\overset{2}{\underset{\text{k}=1}{\sum }}\gamma_{\text{k}}{\left(\text{x}\times {\text{z}}_{\text{k}}\right)}_{\text{i}}+\varepsilon_{\text{i}}$$

where for the ith observation, x_i_ is a binary fixed effect representing genotype (only two genotypes are present in the dataset, homozygous or heterozygous), and z_k_i__ is a categorical fixed effect representing time in sustained hypoxia; when k = 1 it refers to the time point at 4 h of sustained hypoxia and when k = 2 it refers to the time point at 8 h of sustained hypoxia. A similar model was used for the genotype at *EPAS1* where x_i_ was a categorical fixed effect representing the three genotypes (i.e., minor homozygote, heterozygote, or major homozygote). ã ∼ N (α, σ_α_^2^) is a random variable reflecting intervolunteer variability and ε_i_ ∼ N(0, σ^2^) is the interobservation error.

For the analysis examining the influence of ethnicity on the induction of HIF-target genes by graded hypoxia in PBL, the following linear mixed-effect model was used:

$${\text{y}}_{\text{i}}=ã\;+\beta {\text{x}}_{\text{i}}+\gamma {\text{z}}_{\text{i}}+\delta {\text{z}}_{\text{i}}^{3}+\zeta {\left(\text{x}\times \text{z}\right)}_{\text{i}}+\overset{4}{\underset{\text{k}=2}{\sum }}{ñ\;}_{\text{k}}{\text{w}}_{{\text{k}}_{\text{i}}}+\overset{4}{\underset{\text{k}=2}{\sum }}\theta_{\text{k}}{\left(\text{z}\times {\text{w}}_{\text{k}}\right)}_{\text{i}}+\varepsilon_{\text{i}}$$

where for the ith observation, x_i_ represents ethnicity and is a binary fixed effect and z_i_ is a continuous variable representing log (oxygen concentration) and w_k_i__ is a categorical fixed effect representing gene; k = 2 refers to gene adrenomedullin, k = 3 refers to gene aldolase C, and k = 4 refers to gene *P4HA1* with the reference gene being VEGFA. ε_i_ ∼ N(0, σ^2^) is the interobservation error. ã ∼ N (α, σ_α_^2^) and η̃_k_ ∼ N(η_k_, σ_η_k__^2^) are random variables reflecting intervolunteer variability for different genes. In this analysis, the dependent variable, y_i_, represents ΔΔCT.
